# *FUM* Gene Expression Profile and Fumonisin Production by *Fusarium verticillioides* Inoculated in *Bt* and Non-*Bt* Maize

**DOI:** 10.3389/fmicb.2015.01503

**Published:** 2016-01-06

**Authors:** Liliana O. Rocha, Vinícius M. Barroso, Ludmila J. Andrade, Gustavo H. A. Pereira, Fabiane L. Ferreira-Castro, Aildson P. Duarte, Marcos D. Michelotto, Benedito Correa

**Affiliations:** ^1^Laboratório de Micotoxinas, Departamento de Microbiologia, Instituto de Ciências Biomédicas, Universidade de São PauloSão Paulo, Brazil; ^2^Departamento de Estatística, Centro de Ciências Exatas e de Tecnologia, Universidade Federal de São CarlosSão Carlos, Brazil; ^3^Centro de Grãos e Fibras, Instituto Agronômico de Campinas, Agência Paulista de Tecnologia dos Agronegócios (APTA)Campinas, Brazil

**Keywords:** transgenic corn, mycotoxins, *Fusarium*, gene expression, *Bacillus thuringiensis*

## Abstract

This study aimed to determine the levels of fumonisins produced by *Fusarium verticillioides* and *FUM* gene expression on *Bt* (*Bacillus thuringiensis*) and non-*Bt* maize, post harvest, during different periods of incubation. Transgenic hybrids 30F35 YG, 2B710 Hx and their isogenic (30F35 and 2B710) were collected from the field and a subset of 30 samples selected for the experiments. Maize samples were sterilized by gamma radiation at a dose of 20 kGy. Samples were then inoculated with *F. verticillioides* and analyzed under controlled conditions of temperature and relative humidity for fumonisin B_1_ and B_2_ (FB_1_ and FB_2_) production and *FUM1, FUM3, FUM6, FUM7, FUM8, FUM13, FUM14, FUM15*, and *FUM19* expression. 2B710 Hx and 30F35 YG kernel samples were virtually intact when compared to the non-*Bt* hybrids that came from the field. Statistical analysis showed that FB_1_ production was significantly lower in 30F35 YG and 2B710 Hx than in the 30F35 and 2B710 hybrids (*P* < 0.05). However, there was no statistical difference for FB_2_ production (*P* > 0.05). The kernel injuries observed in the non-*Bt* samples have possibly facilitated *F. verticillioides* penetration and promoted FB_1_ production under controlled conditions. *FUM* genes were expressed by *F. verticillioides* in all of the samples. However, there was indication of lower expression of a few *FUM* genes in the *Bt* hybrids; and a weak association between FB_1_ production and the relative expression of some of the *FUM* genes were observed in the 30F35 YG hybrid.

## Introduction

Maize (*Zea mays* L.) is one of the world’s major agricultural crops serving as a staple food for millions, with an annual average production and consumption of 989.2 million metric tons (Mt) and 953.9 Mt (2013-2014), respectively. Brazil is currently the world’s third largest producer, after United States and China, with an average production of 79.3 Mt over the last year (United States Department of Agriculture [USDA], 2014).

Susceptible to fungal and mycotoxin contamination, numerous toxic fungal secondary metabolites can be found in maize, though, fumonisins occur with greatest frequency. Members of the *Fusarium fujikuroi* species complex, mainly *F. verticillioides* (Sacc.) Nirenberg and *F. proliferatum* (Matsush.) Nirenberg, are capable of producing these mycotoxins consistently (Desjardins, 2006). *F. verticillioides* is one of the most important species associated with maize throughout the world and produces high levels of fumonisins. Contamination with these mycotoxins has a significant impact, compromising the quality of maize products (de la Campa et al., 2005).

Based on chemical structure, fumonisins have been classified into A, B, C, and P groups; the most common are the B analogs and fumonisin B_1_ (FB_1_) is the most prevalent and toxic of the group (Stepien et al., 2011). Fumonisin B_1_ is known to cause toxicity in animals and humans due to the inhibition of sphingolipid metabolism and cell cycle regulation, resulting in diverse and complex effects. Examples include leukoencephalomalacia in horses, pulmonary oedema, and hydrothorax in swine; fumonisin B_1_ is additionally carcinogenic to rodents and exhibits nephrotoxic as well as hepatotoxic activity in rats and rabbits (Desjardins, 2006). Epidemiological studies have suggested that fumonisins may also be associated with oesophageal cancer and neural tube birth defects in humans (Marasas et al., 2004).

In *F. verticillioides*, fumonisin production is regulated by the fumonisin biosynthetic gene cluster (*FUM*), which consists of 16 genes encoding biosynthetic enzymes, regulatory, and transport proteins (Proctor et al., 2013). *FUM1* catalyzes the synthesis of a linear polyketide that forms the backbone structure of fumonisins. *FUM8* encodes an α-oxoamine synthase responsible for the condensation of the linear polyketide with alanine (Proctor et al., 2003, 2013; Lazzaro et al., 2012; Medina et al., 2013). The cluster also encodes a C-3 carbonyl reductase (*FUM13*), and cytochrome P450 oxygenases (*FUM2, FUM3, FUM6, FUM15*; Proctor et al., 2003; Bojja et al., 2004). The genes *FUM7, FUM10, FUM11, FUM14*, and *FUM16* are required for tricarballylic acid esterification. *FUM17* and *FUM18* are longevity assurance factors; and *FUM19* encodes a protein highly similar to ABC multidrug transporters, which can possibly reduce cellular concentration of toxins, therefore conferring self-protection (Proctor et al., 2003; Bojja et al., 2004; Desjardins, 2006). *FUM21* encodes a Zn(II)2Cys6 binuclear DNA-binding transcription factor that positively regulates *FUM* gene expression (Brown et al., 2007). Studies have demonstrated that these genes are correlated with fumonisin production (Lopez-Errasquín et al., 2007; Medina and Magan, 2010; Rocha et al., 2011) and the expression of some mycotoxin genes respond positively to different environmental conditions (Medina and Magan, 2010).

Globally, fumonisin contamination in food is a concern. The FDA (the United States Food and Drug Administration) has established a limit of 3–4 ppm of fumonisin contamination (FB_1_ + FB_2_ + FB_3_) in human foods and 5–100 ppm in animal feeds. The Commission Regulation (EC) of the European Union has established a maximum level of 4 ppm for FB_1_ + FB_2_ in unprocessed maize and 1 ppm in maize and maize-based products intended for human consumption (European Commission Regulation [EC], 2007). However, the legislation is distinct around the world, depending on the concern that the authorities have about the potential toxic effects of fumonisins on animals and their implications for industry (Food and Drug Administration [FDA], 2001; Schatzmayr and Streit, 2013).

Low levels of fumonisins can occur even in intact maize kernels, since *F. verticillioides* is found in both asymptomatic and diseased plants. Environmental conditions, water availability and the genetic background of the plant and the pathogen are significant factors in disease development and mycotoxin production (Oren et al., 2003; Bowers et al., 2013). It has been shown that physically injured kernels increase the propensity for fungal contamination and mycotoxin production (Munkvold et al., 1999; Parsons and Munkvold, 2012; Bowers et al., 2013). Kernel injuries caused by ear feeding insects are particularly important. These pests act as vectors for fungal spores, increasing the severity of fungal disease and mycotoxin production (Dowd, 2000; Wu, 2006; Ferreira-Castro et al., 2012).

The damage caused by European corn borer (ECB, *Ostrinia nubialis* Hübner), Southwestern corn borer (SWCB, *Diatraea grandiosella* Dyar), corn earworm (CEW, *Helicoverpa zea* Boddie) and fall armyworm (FAW, *Spodoptera frugiperda*, J.E. Smith) has been shown to favor mycotoxin contamination in the field and to contribute to mycotoxin accumulation during storage (Sinha, 1994; Dowd, 2000; Wu, 2006). *Bt* (*Bacillus thuringiensis*) hybrids have been effective in reducing injuries by these insects, indirectly controlling plant susceptibility to fungal infection and mycotoxin contamination (Munkvold et al., 1997; Munkvold and Hellmich, 1999; Ostry et al., 2010; Bowers et al., 2013; Foresti et al., 2013)

It is widely known that *Bt* maize presents a lower risk of fumonisin contamination compared with non-*Bt* hybrids when exposed to lepidopteran insects (Duvick, 2001; Pazzi et al., 2006; Abbas et al., 2011; Bowers et al., 2013, 2014). A recent study has also demonstrated that *Bt* maize exhibited reduction in deoxynivalenol and zearalenone contamination in harvested maize kernels (Ostry et al., 2010). Although many studies have shown the relationship between kernel injuries by insects and fumonisin contamination in maize during harvest, none has focused on the fumonisin accumulation in *Bt* and non-*Bt* maize after this period.

The aim of this study was to verify the levels of fumonisins produced by *F. verticillioides* on *Bt* and non-*Bt* maize, post harvest, in different periods of incubation under controlled conditions. The second objective of this study was to compare *FUM* gene expression between *Bt* and non-*Bt* hybrids and to study the association between fumonisin production and *FUM* gene expression by *F. verticillioides* for each of the hybrids.

## Materials and Methods

### Maize Grain Samples

Maize cultivar samples of *Bt* 2B710 Hx and 30F35 YG and their isogenic non-*Bt* 2B710 and 30F35 were provided by the Agronomic Institute of Campinas (Instituto Agronômico de Campinas, APTA-São Paulo, Brazil). These samples were sown in November/2010 and harvested in March/2011 in Cruzália, State of São Paulo, Brazil.

Sampling was conducted according to methodology proposed by Delp et al. (1986) with modifications. *Bt* and non-*Bt* crop fields were sampled by dividing them into four sectors of uniform size containing eight rows each. Ten samples were randomly collected from each sector, for a total of 40 samples per hybrid. The hybrids were harvested manually in the experimental field by the technical support staff of the Agronomic Institute of Campinas. Thirty subsamples for each hybrid were then selected for this experiment, totalling 120 tests.

Ten samples of 30F35 YG (expressing Cry 1Ab protein, equivalent to MON810, Monsanto), 10 samples of 2B710 Hx (expressing Cry 1F protein, equivalent to TC1 507, Dow Agrosciences) corn grains and their respective isogenic hybrids were used for each incubation period (10, 20, and 30 days).

The injuries caused by *S. frugiperda* in 30F35 YG, 2B710 Hx and their isogenic hybrids were previously analyzed from sowing to harvest by The Agronomic Institute of Campinas in the same experimental field used in the current study (Michelotto et al., 2011).

All samples were sterilized by gamma radiation at a dose of 20 kGy to eliminate the natural mycoflora at the Nuclear and Energetic Research Institute (Instituto de Pesquisas Energéticas e Nucleares-IPEN-CNEN/SP). A GammaCell irradiator equipped with a cobalt 60 source was used and a dose rate ranging from 4.74 to 4.84 kGy/h was applied. The mycoflora was verified in order to control the irradiation procedure (Berjak, 1984). Samples were previously analyzed for the presence of fumonisins according to the methodology described by Visconti et al. (2001), because gamma radiation does not eliminate fumonisin from grains (Ferreira et al., 2007). The values (for each sample) were considered to be a basal FB_1_ or FB_2_ contamination, and were used to calculate the final fumonisin concentration produced by *F. verticillioides* after each period of incubation (total concentration minus basal concentration).

### *Fusarium verticillioides* Strain

*Fusarium verticillioides* isolate 13BA was obtained from the culture collection of the Institute of Biomedical Sciences, ICBII, University of São Paulo, Brazil. The isolate was maintained on SNA (Spezieller Nährstoffarmer Agar) by monosporic isolation and it was used in all experiments (Rocha et al., 2011).

Before beginning the experiments, the ability of fumonisin production by *F. verticillioides* strain 13BA was re-evaluated according to methodology proposed by Ross et al. (1990) and Sydenham et al. (1990), with concentrations of 120and 36 μg/g for FB_1_ and FB_2_, respectively.

### Inoculation of Corn Samples with *Fusarium verticillioides* Spore Suspension

The *F. verticillioides* fungal spore suspension was prepared according to Ferreira et al. (2007). The final suspension was adjusted to 5 × 10^5^ spores/mL and inoculated into 5 g of maize bran (previously treated with 20 kGy gamma radiation). This mixture was added to Petri dishes containing 25 g of grains used for fumonisin and *FUM* gene cluster expression analyses. The samples were stored in a plastic container at a relative humidity and temperature controlled by a previously calibrated thermo hygrometer. The container was incubated in a BOD incubator at 25°C and a relative humidity of 97.5 to 99.0% obtained with 30% K_2_SO_4_ solution as described by Winston and Bates (1960) for 10, 20, and 30 days. After each incubation period, 10 samples of each hybrid were analyzed, with 20 g for fumonisin analyses and 5 g for gene expression experiments.

### Determination of Fumonisins

Samples were prepared and extracted according to the methodology proposed by Visconti et al. (2001). Afterwards, fumonisins were mixed with the derivatizing agent ortho-phthaldehyde (OPA; Visconti et al., 2001) and injected into a Shimadzu liquid chromatograph (LC-10AD). Separation was performed on a C-18 reverse phase column (5 ODS-20, 150 mm × 4.6 mm; Phenomenex).

Fumonisins were quantified based on a calibration curve using standard solutions of FB_1_ and FB_2_ (Sigma–Aldrich). The coefficient of correlation was 0.993 for FB_1_ and 0.995 for FB_2_. The limit of quantification (LOQ) for FB_1_ was 0.015 μg/g, with a mean recovery of 92.38% and standard deviation of 13.68% (five replicates). For FB_2_, LOQ was 0.015 μg/g, with a mean recovery of 85.39% and standard deviation of 6.87% (five replicates). LOQ was determined via recovery tests. The lowest validated spike (0.015 μg/g) level with a percentage of recovery between 70 and 120% and a standard variation lower than 20% was defined as the method LOQ in this study (European Commission Regulation [EC], 2007).

The final fumonisin concentration was calculated through the subtraction of the total FB_1_ or FB_2_ for each analyzed sample (basal FB_1_ or FB_2_ concentration plus the concentration of FB_1_ or FB_2_ produced by *F. verticillioides* during the respective incubation periods) minus the basal FB_1_ or FB_2_ contamination of each sample that came from the field.

### *FUM* Gene Cluster Expression

#### Isolation of mRNA and Reverse Transcription

RNA was extracted from 5 g of corn samples corresponding to each inoculation period (10, 20, and 30 days) using the Plant RNA Isolation Aid Kit (Ambion^®^, Life Technologies, Carlsbad, CA, USA) followed by purification utilizing RNAqueous Kit (Ambion^®^, Life Technologies, Carlsbad, CA, USA), according to the manufacturer’s instructions. cDNA was synthesized with the High Capacity cDNA Reverse Transcription kit according to the manufacturer’s instructions (Applied Biosystems, Life Technologies, Carlsbad, CA, USA). cDNA was synthesized in a Gene Amp^®^ PCR System 9700 thermal cycler (Applied Biosystems, Life Technologies) for 1 h at 37°C. The samples were stored at -20°C.

### Quantitative PCR (qPCR)

In this study, the following *FUM* genes were initially tested: *FUM1, FUM2, FUM3, FUM6, FUM7, FUM8, FUM10 FUM13, FUM14, FUM15, FUM19*, and *FUM21.* Primers were designed in the program Primer 3^1^ from the reference sequences AF155773 (Proctor et al., 1999) and U27303/EU430619 (Yan and Dickman, 1996; Visentin et al., 2009) deposited at the National Centre for Biotechnology Information-NCBI^2^. *FUM1, FUM19*, and *TUB* primers were obtained from the study of Lopez-Errasquín et al. (2007; **Table 1**). The specificity of the primer sequences was verified through BLAST tool in NCBI.

**Table 1:** qPCR primers used to evaluate *FUM* gene expression by *Fusarium verticillioides* in maize grains.

Locus	Primer sequence (5′–3′)	Fragment size (bp)	Reference/NCBI accession number
*FUM1F* *FUM1R*	GAGCCGAGTCAGCAAGGATT AGGGTTCGTGAGCCAAGGA	90	Lopez-Errasquín et al., 2007
*FUM3F* *FUM3R*	CTTGGCGGTGCCCATACTA GGACCAAGAGCGTGGATG	60	This study/AF155773
*FUM6F* *FUM6R*	GATAGACTCGGGGCTGAGA AGCTCGCCGACAGAATC	100	This study/AF155773
*FUM7F* *FUM7R*	CATCGTATCTACATTGTCGCATC TGTACTCTCCAACAATATGAATGAGTC	100	This study/AF155773
*FUM8F* *FUM8R*	CAACAGAAATACGCAATGACG TGCTCGACCACTACATCAGG	99	This study/AF155773
*FUM13F* *FUM13R*	GCCTTTGGTCTTGTTCTCTCA CGTCAATTATTGCCTCTTTCAA	100	This study/AF155773
*FUM14F* *FUM14R*	TAGGTCCAGGTCGAGATGCT GGAAGCCAAGAACCCAATCT	99	This study/AF155773
*FUM15F* *FUM15R*	TGCCATCCAGAATGACGATA GAGTCTCAGGAGAGCGAGGA	94	This study/AF155773
*FUM19F* *FUM19R*	ATCAGCATCGGTAACGCTTATGA CGCTTGAAGAGCTCCTGGAT	88	Lopez-Errasquín et al., 2007
*TUBF* *TUBR*	CCGGTATGGGTACTCTGCTC CTCAACGACGGTGTCAGAGA	95	Lopez-Errasquín et al., 2007
*CALMF* *CALMR*	ACGGTTTCATTTCTGCTGCT TCAGCCTCTCGGATCATCTC	97	This study/AF155773

The Platinum SYBR Green qPCR SuperMix-UDG reagent (Invitrogen, Life Technologies) was used as the reaction mixture, according to the manufacturer’s instructions. Each sample was analyzed in duplicate. An appropriate negative control containing no template was used in all reactions to exclude possible contaminations. The quantification of mRNA was normalized using the average of *TUB* (β-tubulin) and *CALM* (calmodulin) genes (Bustin et al., 2009), with cDNA amplifications ran on the same plate. The following reference genes were tested prior to the experiments: *CALM, TUB, EF-1*α (elongation factor 1 α) and *18s rRNA* (18s ribosomal RNA). The Ct values of *TUB* and *CALM* were similar; therefore, the mean values of these genes were used for normalizing the data.

Relative quantification based on ΔΔCt values was the method of analysis used in this study, since the efficiencies of compatibility tests between the average of the reference genes (*TUB* and *CALM*) and the target gene were similar, with slopes between -0.1 and 0.1 (Pfaﬄ, 2001; Gizinger, 2002; Schmittgen and Livak, 2008; Raymaekers et al., 2009).

For validation of the method, serial dilutions of the target genes and of the reference genes were prepared in triplicate. The primers’ efficiency was calculated based on the curve generated by the software. *FUM2, FUM10*, and *FUM21* were excluded from the analysis since qPCR parameters were not acceptable according to the method of analysis proposed by Pfaﬄ (2001) and Schmittgen and Livak (2008).

### Statistical Analysis

The results were analyzed using Gamlss R 2.9 package and Statistical Analysis Software (SAS) version 9.1. The Gamlss model (5% level of significance) with Weibull distribution was used to evaluate differences of FB_1_ and FB_2_ production between *Bt* and non-*Bt* maize during the period of incubation (10, 20, and 30 days; Rigby and Stasinopoulos, 2005). The Weibull distribution was chosen using quantile residual plots (Dunn and Smyth, 1996). The analysis was conducted based on the overall data (from the first to 30th day). The Gamlss model was used to verify the interaction between hybrids (*Bt* and non-*Bt*) and period of incubation (10, 20, and 30 days). As these interactions were not significant (*P* > 0.05), further analyses based on each period of incubation (10, 20, and 30 days) were not conducted.

The differences of *FUM* gene expression between *Bt* and non-*Bt* maize were evaluated using the Mann–Whitney test, since the data was not normally distributed (5% level of significance) for each period of incubation (Gibbons and Chakraborti, 2003). Pearson’s correlation tests were performed to investigate possible associations between the relative expression levels of *FUM1, FUM3, FUM6, FUM7, FUM8, FUM13, FUM14, FUM15*, and *FUM19* and the production of FB_1_ and FB_2_ by *F. verticillioides* on *Bt* and non-*Bt* samples.

## Results

### Production of FB_1_ and FB_2_ in *Bt* and Non-*Bt* Maize Samples Artificially Contaminated with *F. verticillioides* During the Periods of 10, 20, and 30 days

FB_1_ and FB_2_ were quantified during 10, 20, and 30 days of incubation. The *Bt* hybrid 30F35 YG presented FB_1_ levels between 0.11 to 4.99 μg/g (mean: 2.38 μg/g) and FB_2_ from 0.02 to 2.66 μg/g (mean: 1.53 μg/g). 30F35 hybrid presented FB_1_ levels from 1.49 to 5.34 μg/g (mean: 3.17 μg/g) and FB_2_ from 0.015 to 2.83 μg/g (mean: 1.7 μg/g) (**Table 2**). Statistical analysis showed that FB_1_ contamination was significantly lower in 30F35YG when compared to its isogenic hybrid 30F35 (*P* = 0.0095). However, there was no statistical difference between *Bt* and non-*Bt* maize samples for FB_2_ production (*P* = 0.827). Interaction between hybrids (*Bt* and non-*Bt*) and period of incubation (10, 20, and 30 days) was not significant (*P* > 0.05), therefore there was no evidence that the mean difference of FB_1_ (*P* = 0.26) and FB_2_ (*P* = 0.82) production between *Bt* and non-*Bt* maize changed over the period of incubation.

**Table 2:** Fumonisin production by *F. verticillioides* in 30 maize samples of 30F35 YG, 2B710 Hx (*Bt*), 30F35 and 2B710 (non-*Bt* hybrids) during 10, 20, and 30 days of incubation under temperature of 25°C and humidity of 97.5 to 99.0%.

Period of Incubation	Samples	30F35 YG (*Bt*)		30F35 (non-*Bt*)		2B710 Hx (*Bt*)		2B710 (non-*Bt*)	
		FB_1_ (μg/g)	FB_2_ (μg/g)	FB_1_ (μg/g)	FB_2_ (μg/g)	FB_1_ (μg/g)	FB_2_ (μg/g)	FB_1_ (μg/g)	FB_2_ (μg/g)
10 days	1	2,92^b^	2,26	3,14	2,66	7,65	3,50	8,05	4,59
	2	2,91	2,60^b^	3,78	2,83^b^	6,72	2,61	10,90	5,68
	3	0,99	0,52	1,58	0,63	7,52	3,48	9,36	5,15
	4	1,99	0,60	2,07	0,41^a^	0,19^a^	0,02^a^	7,92	3,68
	5	1,29	2,54	4,64^b^	2,29	8,15	3,83	6,95	3,56
	6	0,32^a^	0,61	2,06	0,78	6,01	6,03	8,98	2,59
	7	0,84	0,37^a^	3,51	1,77	3,58	1,28	6,13	3,11
	8	2,02	0,57	4,19	2,21	7,70	5,68	4,46^a^	2,89
	9	1,24	0,49	1,49^a^	0,53	4,34	3,64	4,90	0,80^a^
	10	2,64	2,29	2,71	2,30	8,24^b^	6,75^b^	11,03^b^	8,31^b^

Mean		^c^1.72 ± 0.29^∗^	1.29 ± 0.31	^d^2.92 ± 0.35	1.64 ± 0.30	^e^6.01 ± 0.82	3.68 ± 0.66	^f^7.87 ± 0.72	4.04 ± 0.65
20 days	11	2,71	1,96	2,86	1,99	8,74	6,56	9,57	5,42
	12	4,99^b^	1,64	5,11^b^	1,64	7,26	5,89	10,34	5,87
	13	4,31	2,66^b^	4,42	2,66^b^	8,49	3,02	9,16	4,36
	14	3,47	2,01	3,76	2,03	7,24	3,68	10,97^b^	6,58^b^
	15	3,62	1,03	4,09	1,07	5,72	2,95	7,52	5,46
	16	3,48	1,40	3,65	1,41	6,11	2,24^a^	5,05^a^	2,46^a^
	17	1,00	1,66	3,12	2,05	7,90	6,10	10,16	5,88
	18	3,03	2,27	3,16	2,27	4,97^a^	3,05	6,83	3,45
	19	0,41^a^	1,17	2,15	1,67	9,00^b^	6,72^b^	10,47	5,22
	20	1,00	0,02^a^	1,84^a^	0,015^a^	5,64	3,98	7,37	4,45

Mean		^c^2.8 ± 0.48	1.58 ± 0.23	^d^3.42 ± 0.32	1.68 ± 0.23	^e^7.11 ± 0.45	4.42 ± 054	^f^8.74 ± 0.61	4.92 ± 0.39
30 days	21	2,87	1,55	2,96	1,55	4,43	3,64	9,24	7,45
	22	0,11^a^	1,60	2,55	1,61	5,55	2,42	9,00	6,32
	23	2,30	0,91^a^	2,45^a^	0,92^a^	5,56	1,55	9,43	7,90^b^
	24	2,89	2,17^b^	2,98	2,17	3,44	2,76	3,33^a^	0,98^a^
	25	2,83	1,88	2,90	1,88	8,43	4,95	11,55^b^	7,47
	26	2,71	1,86	2,82	1,87	9,47	5,49	9,70	3,35
	27	3,72^b^	2,06	4,01	2,08	9,71^b^	6,62	7,86	3,62
	28	2,38	1,51	2,64	1,51	3,72	2,92	9,61	5,21
	29	2,96	1,83	3,15	1,83	2,59^a^	1,50^a^	8,22	2,56
	30	3,49	1,70	5,34^b^	2,28^b^	8,63	6,64^b^	10,73	5,66

Mean		^c^2.63 ± 0.31	1.71 ± 0.11	^d^3.18 ± 0.28	1.77 ± 0.12	^e^6.15 ± 0.85	3.85 ± 0.62	^f^8.87 ± 0.70	5.05 ± 0.74

Mean/total		^c^2,38 ± 0.22	1,53 ± 0.13	^d^3,17 ± 0.18	1,7 ± 0.13	^e^6.58 ± 0.41	3.98 ± 0.36	^f^8.68 ± 0.31	4.67 ± 0.35

*Fumonisin levels were calculated as described in section “Materials and Methods.”*

*^a^Minimum fumonisin level for each 10, 20, and 30 days; ^b^Maximum fumonisin level for each 10, 20, and 30 days; ^∗^Mean ± standard error; ^c,d^Differences of FB_1_ production in 30F35 YG and 30F35, respectively (P < 0.05); ^e,f^Differences of FB_1_ production in 2B710 Hx and 2B710, respectively (P < 0.05).*

2B710 Hx presented levels of FB_1_ and FB_2_ between 0.19–9.71 μg/g (mean: 6.42 μg/g) and 0.02–6.75 μg/g (mean: 3.98 μg/g), respectively. The isogenic 2B710 hybrid presented FB_1_ levels from 3.33–11.55 μg/g (mean: 8.49 μg/g) and FB_2_ from 0.8 to 8.31 μg/g (mean: 4.67 μg/g) (**Table 2**). FB_1_ contamination was significantly lower in 2B710Hx when compared to its isogenic hybrid 2B710 (*P* = 0.0001). There was no statistical difference between *Bt* and non-*Bt* maize samples for FB_2_ production (*P* = 0.382). There was no evidence that the mean difference of FB_1_ (*P* = 0.95) and FB_2_ (*P* = 0.61) production between *Bt* and non-*Bt* maize changed over the period of incubation.

### Optimization of qPCR Reactions and *FUM* Gene Expression in *Bt* and Non-*Bt* Maize Samples Artificially Contaminated with *F. verticillioides* During the Periods of 10, 20, and 30 days

The comparative ΔΔCt method was chosen for *FUM* gene expression analysis, since qPCR efficiencies were similar. ΔΔCt relative quantification is used to compare the gene expression between one sample and another (control). In this study, the control was considered the samples with the lowest Ct value for all of the genes for each hybrid (Supplementary Tables S1 and S2).

The efficiencies (E) obtained in the qPCR analysis were between 78 and 110% for all of the studied genes, with slopes between -4 (*FUM21*) and -3.1 (*FUM8, FUM14, FUM15*) and *r*^2^ from 0.97 (*FUM2, FUM6, FUM10*) to 0.99 (all the other genes; Supplementary Figure S1). According to the European Network of GMO Laboratories, acceptable slopes for standard curve range from -3.1 to -3.6. In this study, *FUM2, FUM10*, and *FUM21* presented values outside this range (European Network of Gmo Laboratories [ENGL], 2008). The melt-curve analysis resulted in one single peak for all of the genes used in the analysis (Supplementary Figure S1).

The average between the Ct values of the reference genes β-tubulin (*TUB*) and *CALM* were selected to normalize the Ct values of the samples. The average of *CALM* and *TUB* showed better repeatability and less variability in Ct values when compared to other tested genes (*EF- 1α* and *18s rRNA*). The ΔCt values were plotted against the log dilutions, and the slopes ranged from -0.24 (*FUM21* in relation to *TUB* and *CALM*) to 0.02 (*FUM 1* in relation to *TUB* and *CALM*). *FUM2, FUM10*, and *FUM21* reactions did not present acceptable validation parameters once slope values for compatibility tests were not within -0.1 and 0.1; therefore they were excluded from the analysis. Even though *r*^2^ for *FUM6* was below 0.99, this gene was not excluded from the analyses, since efficiency and slope values were satisfactory.

Amplification of the *FUM* genes was observed in all of the samples. Statistical analysis showed that there was an indication of higher *FUM1* expression during the three periods of incubation (*P* < 0.02), higher *FUM13* expression during the 10th day of incubation and higher *FUM7, FUM8*, and *FUM14* expressions during the 30th day of incubation (*P* < 0.04) and higher levels of *FUM19* expression in the 20 and 30th days of incubation (*P* < 0.04) in the non-*Bt* 30F35 hybrid. There was an indication of higher *FUM19* expression during the 10th day of incubation in the non-*Bt* 2B710 hybrid (*P* < 0.03).

**FIGURE 1:**
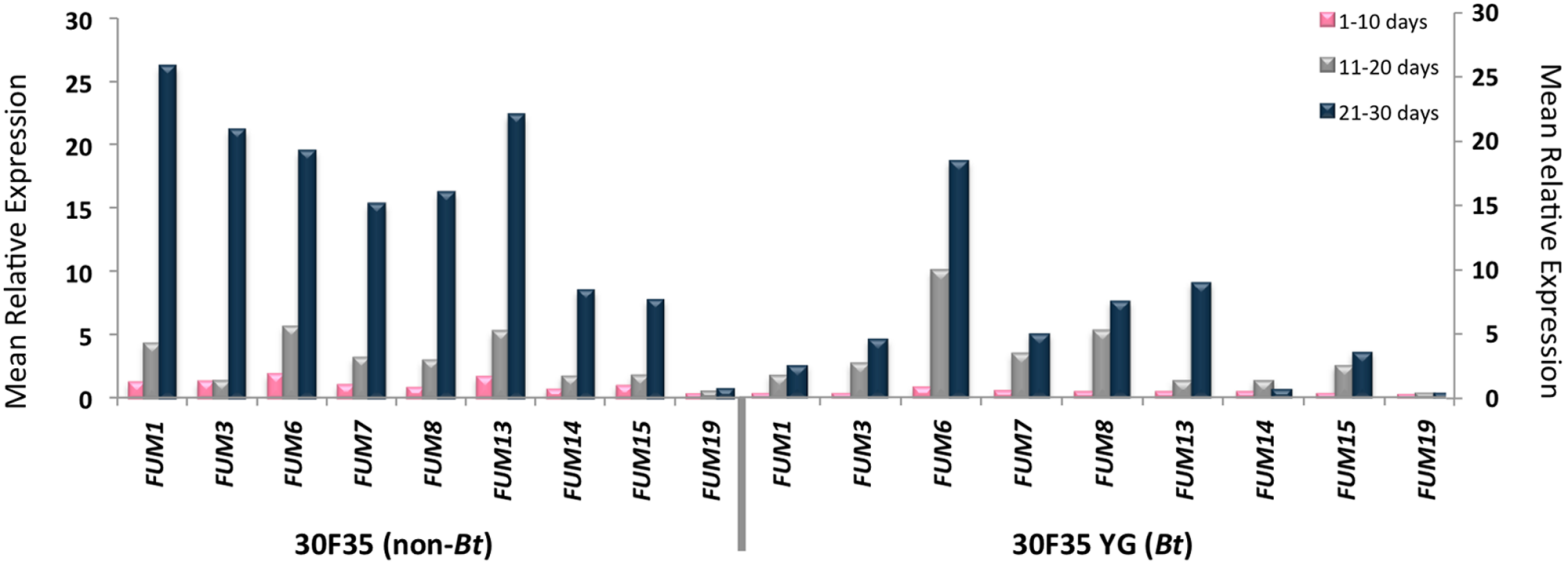
**Mean relative expression of *FUM1, FUM3, FUM6, FUM7, FUM8, FUM13, FUM14, FUM15*, and *FUM19* in 30F35 and 30F35 YG hybrids during 30 days of incubation**.

**FIGURE 2:**
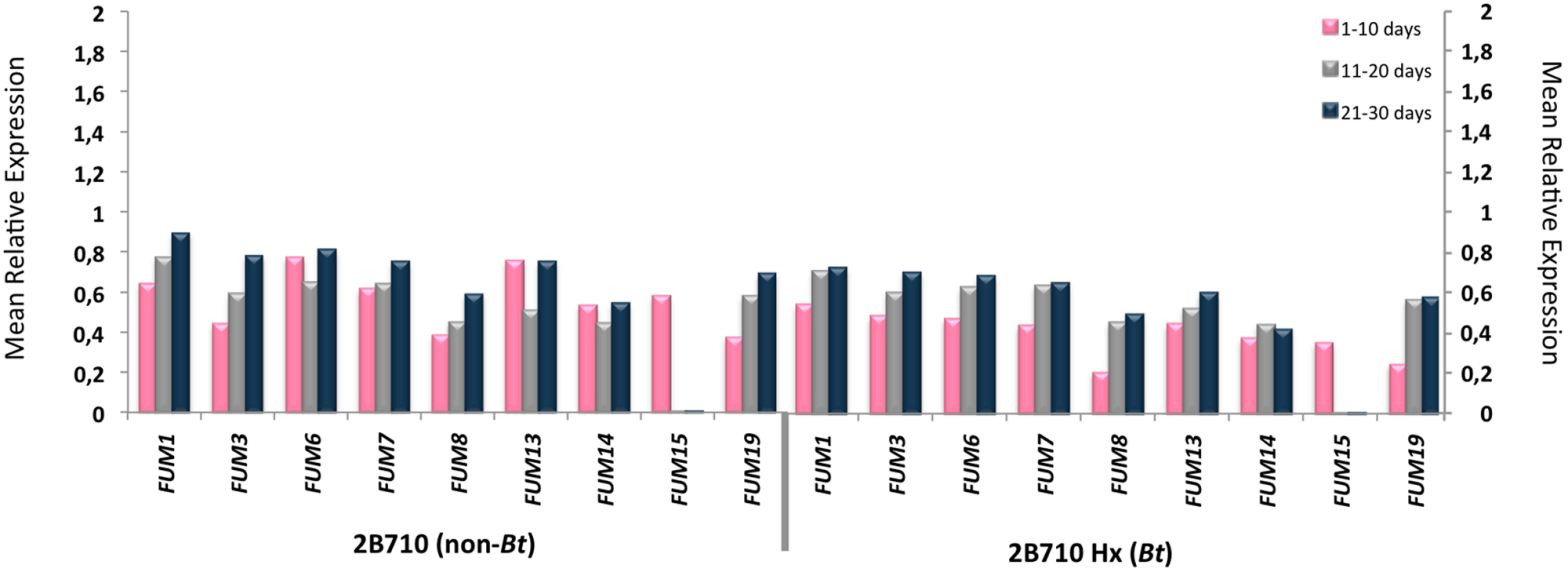
**Mean relative expression of *FUM1, FUM3, FUM6, FUM7, FUM8, FUM13, FUM14, FUM15*, and *FUM19* in 2B710 and 2B710 Hx hybrids during 30 days of incubation**.

There was a weak correlation between FB_1_ production and the expression of *FUM1* (*P* = 0.009, *r* = 0.47), *FUM7* (*P* = 0.017, *r* = 0.43), *FUM8* (*P* = 0.024, *r* = 0.41), *FUM13* (*P* = 0.032, *r* = 0.39), *FUM14* (*P* = 0.001, *r* = 0.57), *FUM15* (*P* = 0.011, *r* = 0.46), and *FUM19* (*P* = 0.009, *r* = 0.47) in corn samples of 30F35 YG hybrid at a 5% significance level. For this hybrid, *FUM19* was also slightly correlated to FB_2_ production (*P* = 0.049, *r* = 0.36). However, there is no evidence of correlation between FB_1_ and FB_2_ production and the studied *FUM* genes for the hybrids 30F35, 2B710, and 2B710 Hx (**Figures 1** and **2**).

## Discussion

Samples representing the non-*Bt* hybrids 2B710 and 30F35 that came from the field presented damaged kernels as a result of insect injuries, which could possibly facilitate fungal penetration and consequently fumonisin production. 2B710 Hx and 30F35 YG samples were virtually intact (Michelotto et al., 2011). It has been shown that wounded plants and seeds facilitate fungal contamination, increasing the chance of diseases and mycotoxin production (Wallin, 1986; Gradziel and Wang, 1994; Mahoney and Rodrigues, 1996; Imathiu et al., 2008; Mohammadi and Hokmabadi, 2010).

The *Bt* hybrids 30F35 YG and 2B710 Hx were chosen due to their worldwide utilization for controlling insect pests. 30F35 YG expresses cry1Ab, an efficient protein for reducing injuries from lepidopteran pests’ feeding (Bowers et al., 2014). Although cry1Ab is not highly effective against CEW (Bowers et al., 2013) and FAW, important lepidopteran insects found in Brazil (Farias et al., 2014), this protein has demonstrated efficacy in preventing insect injuries and therefore aiding in the reduction of fungal contamination and mycotoxin production (Munkvold, 2003; Ostry et al., 2010; Leslie and Logrieco, 2014).

2B710 Hx expresses cry1F protein, and it has been shown to be ineffective against CEW (Bowers et al., 2013), however, hybrids expressing this protein have demonstrated temporary efficacy in controlling FAW in Brazil, since cry1F resistant populations of *S. frugiperda* have emerged from maize field where cry1F protein has been extensively used (Farias et al., 2014). This insect is native to the tropical regions of the western hemisphere from the United States to Argentina. The larvae feed primarily on maize leaf tissue, causing extensive defoliation (Farias et al., 2014); in addition, *S. frugiperda* can migrate to corn ear, causing damage to kernels (Gorman and Kang, 1991), creating infection sites for toxigenic fungi, such as *Fusarium* and *Aspergillus* (Munkvold, 2003, 2014).

Complementary research conducted by Michelotto et al. (2011), using the field and maize samples corresponding to those used in this study, has demonstrated that the damage caused by *S. frugiperda* was significantly lower in 30F35 YG and 2B710 Hx when compared to their respective isogenic hybrids from sowing to harvest. This may explain the pattern of fumonisin production in the samples under controlled conditions, since damaged kernels can promote *F. verticillioides* invasion and therefore facilitate fumonisin production in non-*Bt* maize. In fact, insects are able to cause injuries to the external protection of grains and plant tissues in the field, allowing the fungal hyphae to readily penetrate, have access to nutrients and produce mycotoxins (Wu et al., 2004; Wu, 2006; Jouany, 2007). Since *Bt* maize reduces insect damage and the risk of fungal contamination, it could indirectly control mycotoxin accumulation during storage. However, it is important to emphasize that a deeper investigation regarding the differences between *Bt* and non-*Bt* maize in *F. verticillioides* contamination and fumonisin production as well as the use of different cry proteins to effectively control insect pests are warranted to aid in the management strategies and to better understand the correlation between insect pest control and fungal invasion/mycotoxin production in maize.

The current study also compared the fumonisin production by *F. verticillioides* with *FUM1, FUM3, FUM6, FUM7, FUM8, FUM13, FUM14, FUM15*, and *FUM19* gene expression for each of the studied hybrids, and also compared the *FUM* gene expression between *Bt* and non-*Bt* hybrids.

*FUM* genes were expressed by *F. verticillioides* in all of the corn samples, with an indication of lower gene expression for some of the studied genes in the *Bt* hybrids. There was a weak correlation between FB_1_ and FB_2_ production and the relative expression of some of the studied *FUM* genes were observed in 30F35 YG. Among these genes, *FUM1, FUM14*, and *FUM19* were better correlated with fumonisin production. Interestingly, it was observed differences in the expression of these same genes in *Bt* and non-*Bt* hybrids.

Studies have shown a correlation between some of the *FUM* genes and fumonisin production (Lopez-Errasquín et al., 2007; Jurado et al., 2010; Rocha et al., 2011; Lazzaro et al., 2012; Medina et al., 2013). *FUM19* encodes a protein that is highly similar to the ATP-binding cassette of multidrug resistance transporters, which act as eﬄux pumps to reduce cellular concentrations of toxins and thereby confer protection (Desjardins, 2006). Although Lopez-Errasquín et al. (2007), Jurado et al. (2010) and Rocha et al. (2011), have found a positive correlation between *FUM19* and fumonisin production, our results have shown a weak association between this gene expression and fumonisin production in 30F35 YG hybrid. *FUM14* also had a weak association with fumonisin production; this gene encodes a protein involved in the formation of tricarballylic ester function. Indeed, a previous study has demonstrated that *FUM14*, as well as *FUM1* and *FUM19* exhibit a significant effect on the synthesis of FB_1_ and FB_2_ (Medina et al., 2013). Further studies regarding the expression of these genes should be conducted to verify whether or not they could be useful for diagnostics.

It is relevant to emphasize that previous studies have used synthetic media to produce fumonisins and analyze *FUM* gene expression (Lopez-Errasquín et al., 2007; Jurado et al., 2010; Rocha et al., 2011; Lazzaro et al., 2012; Medina et al., 2013), which increases the quality and concentration of RNA. Fungal RNA extraction from maize can be limited, once kernel consists of 90% starch and 10% proteins (Gibbon and Larkins, 2005). Even though the experimental Petri dishes containing maize grains were covered in *F. verticillioides* mycelium, the final RNA consisted of plant and fungal RNA, which likely decreased the performance of qPCR reactions, thereby interfering in the correlation between *FUM* gene cluster and fumonisin production. The rationale for isolating fungal RNA from maize grains is due to the fact that *F. verticillioides* can persist in undamaged plants and produce fumonisins; thus, identifying an adequate genetic marker for assessing fumonisin production during interaction between *F. verticillioides* and symptomless plants could aid in the prevention of fumonisin accumulation in the field and storage (Presello et al., 2007; Duncan and Howard, 2010; Bowers et al., 2013).

## Conclusion

Non-*Bt* maize samples 30F35 and 2B710 presented damaged kernels caused by insects in the field. In this study, wounded grains have possibly facilitated *F. verticillioides* penetration, leading to higher fumonisin production in non-*Bt* hybrids during the period of incubation due to the increase of fungal biomass. Insects cause physical damage, disruption of nutrients and grain injuries to several crops. Management of insects through the use of *Bt* maize can greatly reduce insect damage, thus indirectly controlling fungal and mycotoxin contamination in the field and the accumulation of mycotoxins during storage. Despite the known effect of *Bt* maize on fumonisin content in the field, due to the control of insect pests, there is a lack of information regarding the accumulation of mycotoxins in *Bt* maize during a post-harvest period. Highlighting that, further investigations using different *Bt* hybrids could possibly aid in the controlling strategies. The gene expression analyses demonstrated that further studies should be conducted to determine an acceptable qualitative genetic marker for identifying fumonisin production in early stages of infection by *F. verticillioides* in maize.

## Author Contributions

LR, BC, and AD conceived the study. AD and MM provided the maize samples. MM performed field sampling and initial tests for grain damage. VB and LA conducted fumonisin analysis. FF-C conducted gene expression tests. GP performed statistical analyses. LR and GP analyzed the overall data for the manuscript. LR wrote the manuscript. All authors approved the final version of the manuscript.

## Conflict of Interest Statement

The authors declare that the research was conducted in the absence of any commercial or financial relationships that could be construed as a potential conflict of interest.

## Footnotes

1 http://frodo.wi.mit.edu/primer3/

2 http://www.ncbi.nlm.nih.gov/

## Supplementary material

FIGURE S1 **Optimization of qPCR reactions. (A)** *FUM1*, **(B)** *FUM3*, **(C)** *FUM6*, **(D)** *FUM7*, **(E)** *FUM8*, **(F)** *FUM13*, **(G)** *FUM14*, **(H)** *FUM15*, and **(i)** *FUM19* and reference genes [**(J)** *TUB* and **(K)** *CALM*]. Melt- curve, slope, *r*^2^, and efficiency results for each of the studied genes. Curves were generated by four dilutions of total fungal RNA (10-fold dilutions for each point of the curve) extracted from maize grains contaminated with *Fusarium verticillioides* mycelium. Five replicates were tested for each dilution, and the slope, *r*^2^ and efficiencies were calculated.
