# Supplementary figures and images for: FUM Gene Expression Profile and Fumonisin Production by Fusarium verticillioides Inoculated in Bt and Non-Bt Maize
Source: Front Microbiol. 2016 Jan 6;6:1503. doi: 10.3389/fmicb.2015.01503 (PMC4701941; doi:10.3389/fmicb.2015.01503)

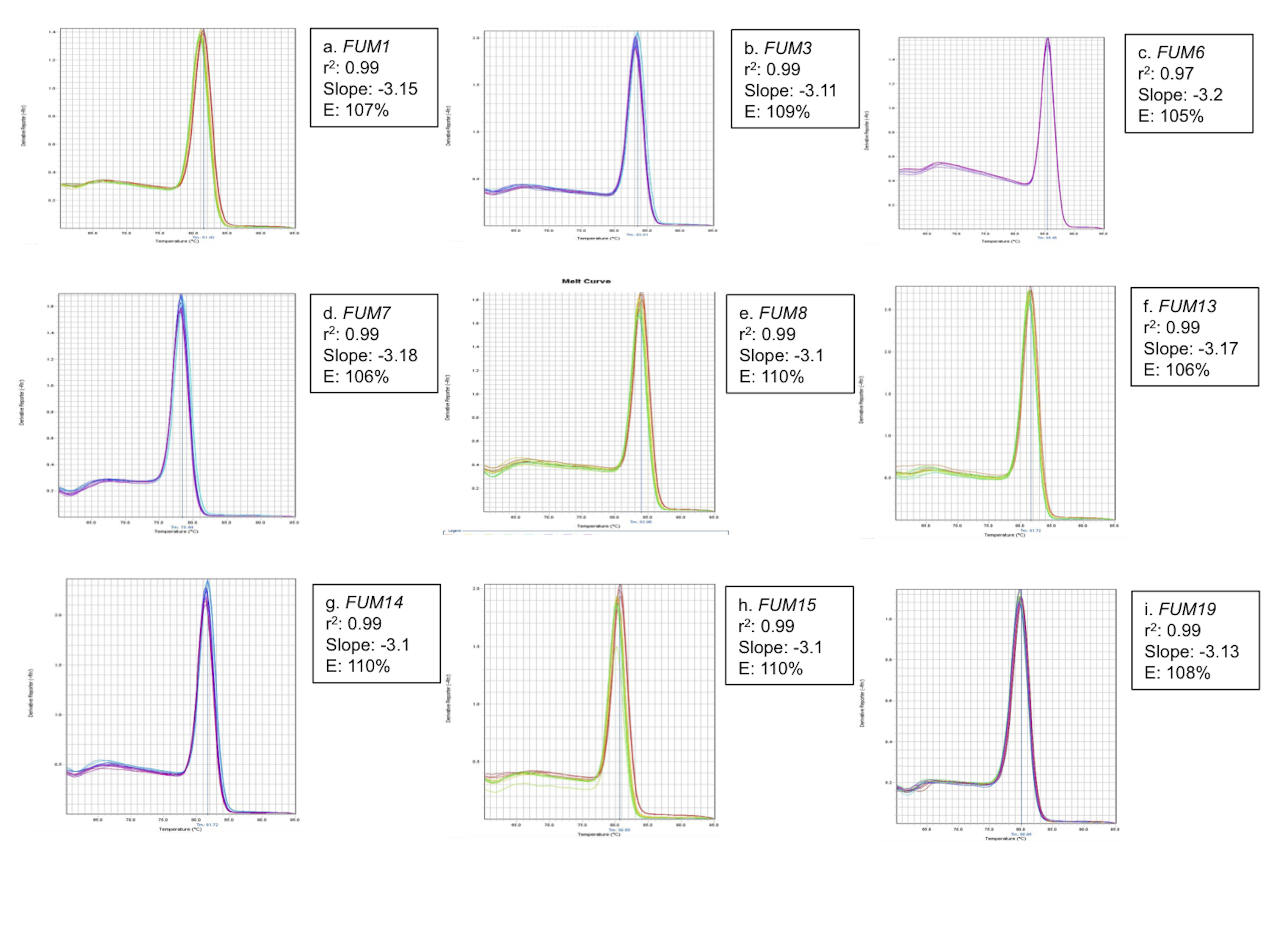

Supplement: FIGURE S1 — Optimization of qPCR reactions. (A) FUM1, (B) FUM3, (C) FUM6, (D) FUM7, (E) FUM8, (F) FUM13, (G) FUM14, (H) FUM15, and (i) FUM19 and reference genes [(J) TUB and (K) CALM]. Melt- curve, slope, r2, and efficiency results for each of the studied genes. Curves were generated by four dilutions of total fungal RNA (10-fold dilutions for each point of the curve) extracted from maize grains contaminated with Fusarium verticillioides mycelium. Five replicates were tested for each dilution, and the slope, r2 and efficiencies were calculated. [file Image_1.TIFF]
