# Supplementary material for: FUM Gene Expression Profile and Fumonisin Production by Fusarium verticillioides Inoculated in Bt and Non-Bt Maize
Source: Front Microbiol. 2016 Jan 6;6:1503. doi: 10.3389/fmicb.2015.01503 (PMC4701941; doi:10.3389/fmicb.2015.01503)
Supplement: Supplementary file 2 [file Table_1.DOCX]

Supplementary Table S1. Fumonisin production by *F. verticillioides* and *FUM1, FUM3, FUM6, FUM7, FUM8, FUM13, FUM14, FUM15* and *FUM19* relative gene expression in 30F35 YG and 30F35 hybrids during 30 days of incubation. The expression levels represent the times that *FUM* genes were expressed in each sample compared to sample 10 (30F35 YG hybrid) and sample 4 (30F35 hybrid) (set at 1.0)

| **Period of incubation** | **Samples** | **FB_1_**  **(µg/Kg)** | **FB_2_**  **(µg/Kg)** | ***FUM1* ^a^** | ***FUM3*** | ***FUM6*** | ***FUM7* ^a^** | ***FUM8* ^a^** | ***FUM13* ^a^** | ***FUM14* ^a^** | ***FUM15*^a^** | ***FUM19 ^b^*** | **FB_1_**  **(µg/Kg)** | **FB_2_**  **(µg/Kg)** | ***FUM1*** | ***FUM3*** | ***FUM6*** | ***FUM7*** | ***FUM8*** | ***FUM13*** | ***FUM14*** | ***FUM15*** | ***FUM19*** |
| --- | --- | --- | --- | --- | --- | --- | --- | --- | --- | --- | --- | --- | --- | --- | --- | --- | --- | --- | --- | --- | --- | --- | --- |
|  | | **30F35 YG (*Bt*)** | | | | | | | | | | | **30F35 (non- *Bt*)** | | | | | | | | | | |
| **1-10 days** | **1** | **2,92** | **2,26** | 0,255 | 0,211 | 0,736 | 0,414 | 0,453 | 0,394 | 0,283 | 0,248 | 0,343 | **3,14** | **2,66** | 2,805 | 1,292 | 5,163 | 2,884 | 2,232 | 5,457 | 1,535 | 2,294 | 0,097 |
|  | **2** | **2,91** | **2,6** | 0,264 | 0,239 | 0,668 | 0,420 | 0,432 | 0,469 | 0,239 | 0,332 | 0,423 | **3,78** | **2,83** | 1,775 | 2,204 | 2,511 | 1,422 | 0,645 | 2,294 | 0,706 | 1,273 | 0,106 |
|  | **3** | **0,99** | **0,52** | 0,246 | 0,250 | 0,811 | 0,417 | 0,476 | 0,417 | 0,227 | 0,268 | 0,091 | **1,58** | **0,63** | 1,680 | 1,972 | 2,786 | 1,273 | 0,645 | 1,364 | 0,594 | 0,828 | 0,052 |
|  | **4^d^** | **1,99** | **0,6** | 0,394 | 0,472 | 1,703 | 0,863 | 1,147 | 0,888 | 0,510 | 0,590 | 0,162 | **2,07** | **0,41** | 1 | 1 | 1 | 1 | 1 | 1 | 1 | 1 | 1 |
|  | **5** | **1,29** | **2,54** | 0,106 | 0,175 | 0,314 | 0,213 | 0,269 | 0,241 | 0,129 | 0,173 | 0,201 | **4,64** | **2,29** | 0,432 | 1,705 | 0,554 | 0,378 | 0,222 | 0,476 | 0,114 | 0,105 | 0,623 |
|  | **6** | **0,32** | **0,61** | 0,101 | 0,142 | 0,180 | 0,100 | 0,102 | 0,192 | 0,262 | 0,185 | 0,199 | **2,06** | **0,78** | 0,339 | 0,953 | 0,368 | 0,279 | 0,293 | 0,1 | 0,1 | 0,1 | 0,623 |
|  | **7** | **0,84** | **0,37** | 0,113 | 0,130 | 0,432 | 0,211 | 0,173 | 0,172 | 1 | 0,139 | 0,196 | **3,51** | **1,77** | 0,834 | 1,050 | 0,900 | 0,570 | 0,543 | 1,503 | 0,238 | 0,435 | 0,275 |
|  | **8** | **2,02** | **0,57** | 0,408 | 0,447 | 1,282 | 0,762 | 0,423 | 0,456 | 0,472 | 0,389 | 0,082 | **4,19** | **2,21** | 1,124 | 1,133 | 1,715 | 0,971 | 0,857 | 1,273 | 0,513 | 0,673 | 0,051 |
|  | **9** | **1,24** | **0,49** | 0,510 | 0,546 | 1,514 | 1,041 | 0,731 | 0,906 | 0,757 | 0,550 | 0,216 | **1,49** | **0,53** | 1,318 | 1,292 | 1,374 | 0,882 | 0,682 | 1,247 | 0,543 | 0,528 | 0,546 |
|  | **10^c^** | **2,64** | **2,29** | 1 | 1 | 1 | 1 | 1 | 1 | 1 | 1 | 1 | **2,71** | **2,3** | 1,383 | 0,438 | 2,511 | 0,590 | 1,345 | 2,408 | 0,778 | 1,318 | 0,297 |
| **11-20 days** | **11** | **2,71** | **1,96** | 2,082 | 2,025 | 8,622 | 3,454 | 5,345 | 4,850 | 2,617 | 3,290 | 0,389 | **2,86** | **1,99** | 3,701 | 1,231 | 5,728 | 2,408 | 1,956 | 6,765 | 1,877 | 2,343 | 0,109 |
|  | **12** | **4,99** | **1,64** | 3,048 | 2,672 | 12,110 | 4,621 | 7,558 | 7,879 | 3,336 | 4,402 | 0,746 | **5,11** | **1,64** | 3,027 | 0,812 | 5,930 | 2,528 | 3,112 | 6,013 | 1,462 | 2,171 | 0,444 |
|  | **13** | **4,31** | **2,66** | 1,171 | 1,108 | 3,753 | 2,082 | 3,454 | 3,290 | 1,545 | 1,813 | 0,082 | **4,42** | **2,6** | 4,784 | 0,717 | 3,912 | 4,107 | 5,728 | 1,984 | 1,634 | 1,309 | 0,4 |
|  | **14** | **3,47** | **2,01** | 1,179 | 1,535 | 6,718 | 2,747 | 3,406 | 3,832 | 1,691 | 2,216 | 0,271 | **3,76** | **2,03** | 3,359 | 0,790 | 4,463 | 2,617 | 3,313 | 4,311 | 1,462 | 2,247 | 0,346 |
|  | **15** | **3,62** | **1,03** | 1,611 | 1,813 | 7,454 | 2,965 | 5,163 | 4,918 | 1,970 | 2,904 | 0,195 | **4,09** | **1,07** | 2,278 | 2,266 | 3,027 | 1,327 | 0,586 | 5,056 | 1,264 | 1,056 | 0,554 |
|  | **16** | **3,48** | **1,4** | 2,068 | 3,967 | 5,163 | 4,750 | 6,013 | 7,879 | 0,486 | 2,654 | 0,301 | **3,65** | **1,41** | 6,097 | 1,516 | 7,052 | 4,463 | 2,945 | 7,934 | 2,728 | 2,232 | 0,692 |
|  | **17** | **1** | **1,66** | 1,864 | 3,805 | 16,890 | 4,402 | 6,718 | 8,271 | 0,438 | 2,844 | 0,266 | **3,12** | **2,05** | 6,812 | 1,141 | 12,110 | 5,382 | 4,718 | 7,352 | 3,006 | 2,635 | 0,469 |
|  | **18** | **3,03** | **2,27** | 3,091 | 7,250 | 29,002 | 6,907 | 11,143 | 13,251 | 0,752 | 4,311 | 0,366 | **3,16** | **2,27** | 3,382 | 1,569 | 2,216 | 2,053 | 1,309 | 1,589 | 0,397 | 0,126 | 0,550 |
|  | **19** | **0,41** | **1,17** | 0,650 | 1,589 | 6,055 | 1,645 | 2,039 | 2,459 | 0,171 | 0,004 | 0,088 | **2,15** | **1,67** | 7,200 | 1,133 | 9,177 | 4,987 | 4,402 | 9,836 | 2,563 | 2,053 | 0,466 |
|  | **20** | **1** | **0.02** | 0,641 | 1,108 | 4,918 | 1,063 | 2,171 | 1,656 | 0,174 | 0,546 | 0,073 | **1,84** | **0,02** | 1,680 | 1,705 | 1,751 | 1,188 | 0,696 | 0,869 | 0,363 | 0,145 | 0,346 |
| **21-30 days** | **21** | **2,87** | **1,55** | 1,851 | 3,779 | 14,909 | 3,912 | 6,580 | 8,743 | 0,510 | 3,526 | 0,250 | **2,96** | **1,55** | 1,393 | 29,144 | 26,810 | 23,072 | 25,073 | 41,015 | 10,254 | 7,352 | 0,762 |
|  | **22** | **0,11** | **1,6** | 1,589 | 3,625 | 13,251 | 2,945 | 5,495 | 6,580 | 0,363 | 3,526 | 0,222 | **2,55** | **1,61** | 20,085 | 26,456 | 25,6 | 9,634 | 10,689 | 19,134 | 6,013 | 5,021 | 0,875 |
|  | **23** | **2,3** | **0,91** | 0,958 | 1,956 | 8,271 | 2,310 | 3,134 | 3,478 | 0,217 | 1,355 | 0,142 | **2,45** | **0,92** | 3,832 | 6,489 | 16,4 | 2,359 | 2,359 | 2,986 | 1,204 | 0,554 | 0,673 |
|  | **24** | **2,89** | **2,17** | 2,326 | 4,022 | 18,740 | 4,850 | 7,825 | 10,914 | 0,406 | 4,107 | 0,366 | **2,98** | **2,17** | 43,655 | 24,882 | 22,758 | 19,401 | 20,225 | 26,687 | 8,865 | 4,193 | 0,469 |
|  | **25** | **2,83** | **1,88** | 1,374 | 2,563 | 10,838 | 2,825 | 3,967 | 6,812 | 0,329 | 2,156 | 0,172 | **2,9** | **1,88** | 26,873 | 21,5 | 17,741 | 14,104 | 15,868 | 24,052 | 9,114 | 6,534 | 0,762 |
|  | **26** | **2,71** | **1,86** | 3,967 | 7,250 | 32,180 | 7,717 | 15,117 | 19,134 | 0,958 | 7,934 | 0,503 | **2,82** | **1,87** | 9,701 | 4,621 | 17,451 | 6,139 | 5,495 | 1,956 | 1,715 | 0,459 | 0,479 |
|  | **27** | **3,72** | **2,06** | 3,940 | 7,250 | 38,802 | 8,045 | 10,689 | 16,890 | 1,221 | 6,534 | 0,463 | **4,01** | **2,08** | 34,489 | 21,463 | 15,495 | 18,482 | 21,231 | 32,856 | 13,069 | 7,352 | 1,006 |
|  | **28** | **2,38** | **1,51** | 3,091 | 7,250 | 24,388 | 6,765 | 8,865 | 2,884 | 0,659 | 1,691 | 0,4 | **2,64** | **1,51** | 29,611 | 19,817 | 19,644 | 16,773 | 15,012 | 13,069 | 5,382 | 2,476 | 1,345 |
|  | **29** | **2,96** | **1,83** | 4,918 | 5,971 | 16,090 | 8,045 | 10,689 | 11,616 | 0,784 | 6,055 | 0,682 | **3,15** | **1,83** | 27,060 | 22,767 | 16,809 | 15,979 | 15,117 | 12,712 | 9,701 | 6,907 | 0,255 |
|  | **30** | **3,49** | **1,7** | 1,255 | 2,156 | 9,904 | 2,425 | 3,625 | 3,967 | 0,318 | 1,877 | 0,164 | **5,34** | **2,28** | 64,807 | 34,410 | 15,783 | 26,687 | 30,655 | 48,438 | 19,401 | 12,712 | 0,219 |

^a^ Relative gene expression levels that correlate to FB_1_ production. ^a^ *FUM1, FUM7, FUM8, FUM13, FUM14, FUM15* and *FUM19* x FB_1_ (30F35 YG): p-values = 0.009, 0.017, 0.024, 0.032, 0.001, 0.011 and 0.009, respectively.

^b^ Relative gene expression levels that correlate to FB_1_ and FB_2_ production. *FUM19* x FB_1_ and FB_2_ (30F35 YG): p-values = 0.009 and 0.049, respectively;

^c^ Calibrator strain used for relative quantification of *FUM* genes in 30F35 YG hybrid;

^d^ Calibrator strain used for relative quantification of *FUM* genes in 30F35 hybrid.
