# Supplementary material for: FUM Gene Expression Profile and Fumonisin Production by Fusarium verticillioides Inoculated in Bt and Non-Bt Maize
Source: Front Microbiol. 2016 Jan 6;6:1503. doi: 10.3389/fmicb.2015.01503 (PMC4701941; doi:10.3389/fmicb.2015.01503)
Supplement: Supplementary file 3 [file Table_2.DOCX]

Supplementary Table S2. Fumonisin production by *F. verticillioides* and *FUM1, FUM3, FUM6, FUM7, FUM8, FUM13, FUM14, FUM15* and *FUM19* relative gene expression in 2B710 Hx and 2B710 hybrids during 30 days of incubation. The expression levels represent the times that *FUM* genes were expressed in each sample compared to sample 8 (2B710 Hx hybrid) and sample 18 (2B710 hybrid) (set at 1.0).

| **Period of**  **Incubation** | **Samples** | **FB_1_**  **(µg/Kg)** | **FB_2_**  **(µg/Kg)** | ***FUM1*** | ***FUM3*** | ***FUM6*** | ***FUM7*** | ***FUM8*** | ***FUM13*** | ***FUM14*** | ***FUM15*** | ***FUM19*** | **FB_1_**  **(µg/Kg)** | **FB_2_**  **(µg/Kg)** | ***FUM1*** | ***FUM3*** | ***FUM6*** | ***FUM7*** | ***FUM8*** | ***FUM13*** | ***FUM14*** | ***FUM15*** | ***FUM19*** |
| --- | --- | --- | --- | --- | --- | --- | --- | --- | --- | --- | --- | --- | --- | --- | --- | --- | --- | --- | --- | --- | --- | --- | --- |
|  | | **2B710 HX (*Bt*)** | | | | | | | | | | | **2B710 (non- *Bt*)** | | | | | | | | | | |
| **1-10 days** | **1** | **7,65** | **3,5** | 0,593 | 0,567 | 0,973 | 0,602 | 0,272 | 0,812 | 0,444 | 0,816 | 0,230 | **8,05** | **4,59** | 0,713 | 0,595 | 0,874 | 0,407 | 0,248 | 0,775 | 0,716 | 0,781 | 0,394 |
|  | **2** | **6,72** | **2,61** | 0,984 | 0,679 | 0,693 | 0,557 | 0,237 | 0,812 | 0,554 | 0,007 | 0,214 | **10,9** | **5,68** | 0,555 | 0,785 | 0,860 | 0,563 | 0,267 | 0,982 | 0,443 | 0,947 | 0,318 |
|  | **3** | **7,52** | **3,48** | 0,947 | 0,590 | 0,691 | 0,303 | 0,247 | 0,904 | 0,442 | 0,846 | 0,241 | **9,36** | **5,15** | 0,580 | 0,699 | 0,579 | 0,478 | 0,288 | 0,784 | 0,553 | 0, 789 | 0,383 |
|  | **4** | **0,19** | **0,02** | 0,560 | 0,605 | 0,702 | 0,527 | 0,258 | 0,566 | 0,557 | 0,742 | 0,243 | **7,92** | **3,68** | 0,687 | 0,594 | 0,952 | 0,526 | 0,286 | 0,845 | 0,554 | 0,755 | 0,392 |
|  | **5** | **8,15** | **3,83** | 0,469 | 0,589 | 0,78 | 0,477 | 0,237 | 0,636 | 0,443 | 0,921 | 0,279 | **6,95** | **3,56** | 0,394 | 0,346 | 0,868 | 0,391 | 0,217 | 0,759 | 0,591 | 0,677 | 0,357 |
|  | **6** | **6,01** | **6,03** | 0,500 | 0,595 | 0,754 | 0,413 | 0,252 | 0,683 | 0,726 | 0,004 | 0,197 | **8,98** | **2,59** | 0,574 | 0,234 | 0,710 | 0,742 | 0,301 | 0,781 | 0,859 | 0,526 | 0,337 |
|  | **7** | **3,58** | **1,28** | 0,507 | 0,696 | 0,629 | 0,528 | 0,243 | 0,617 | 0,456 | 0,691 | 0,233 | **6,13** | **3,11** | 0,538 | 0,146 | 0,770 | 0,631 | 0,222 | 0,916 | 0,813 | 0,720 | 0,389 |
|  | **8 ^a^** | **7,7** | **5,68** | 1 | 1 | 1 | 1 | 1 | 1 | 1 | 1 | 1 | **4,46** | **2,89** | 0,768 | 0,305 | 0,64 | 0,901 | 0,616 | 0,793 | 0,331 | 0,807 | 0,313 |
|  | **9** | **4,34** | **3,64** | 0,818 | 0,590 | 0,219 | 0,803 | 0,298 | 0,0013 | 0,552 | 0,0054 | 0,187 | **4,9** | **0,8** | 0,829 | 0,250 | 0,72 | 0,797 | 0,641 | 0,365 | 0,394 | 0,00467 | 0,391 |
|  | **10** | **8,24** | **6,75** | 0,898 | 0,686 | 0,014 | 0,859 | 0,31 | 0,117 | 0,141 | 0,0318 | 0,973 | **11,03** | **8,31** | 0,765 | 0,457 | 0,74 | 0,698 | 0,741 | 0,537 | 0,045 | 0,0021 | 0,442 |
| **11-20 days** | **11** | **8,74** | **6,56** | 0,783 | 0,687 | 0,569 | 0,781 | 0,517 | 0,445 | 0,447 | 0,0028 | 0,693 | **9,57** | **5,42** | 1,140 | 0,557 | 0,75 | 0,956 | 0,581 | 0,080 | 0,409 | 0,00287 | 0,461 |
|  | **12** | **7,26** | **5,89** | 0,966 | 0,687 | 0,774 | 0,729 | 0,425 | 0,641 | 0,428 | 0,0015 | 0,691 | **10,34** | **5,87** | 0,765 | 0,653 | 0,197 | 0,718 | 0,704 | 0,586 | 0,590 | 0,00450 | 0,594 |
|  | **13** | **8,49** | **3,02** | 0,909 | 0,697 | 0,459 | 0,760 | 0,417 | 0,598 | 0,447 | 0,0029 | 0,676 | **9,16** | **4,36** | 0,822 | 0,769 | 0,44 | 0,696 | 0,599 | 0,771 | 0,357 | 0,00107 | 0,67 |
|  | **14** | **7,24** | **3,68** | 1,015 | 0,686 | 0,728 | 1 | 0,518 | 0,630 | 0,209 | 0,0022 | 0,646 | **10,97** | **6,58** | 0,816 | 0,703 | 0,78 | 0,692 | 0,45 | 0,552 | 0,431 | 0,00576 | 0,762 |
|  | **15** | **5,72** | **2,95** | 0,850 | 0,629 | 0,789 | 0,886 | 0,570 | 0,619 | 0,543 | 0,0020 | 0,504 | **7,52** | **5,46** | 0,816 | 0,709 | 0,87 | 0,692 | 0,44 | 0,559 | 0,431 | 0,00747 | 0,618 |
|  | **16** | **6,11** | **2,24** | 0,794 | 0,796 | 0,678 | 0,604 | 0,521 | 0,546 | 0,573 | 0,0089 | 0,694 | **5,05** | **2,46** | 0,812 | 0,556 | 0,87 | 0,79 | 0,44 | 0,559 | 0,601 | 0,00747 | 0,619 |
|  | **17** | **7,9** | **6,1** | 0,789 | 0,644 | 0,890 | 0,69 | 0,467 | 0,615 | 0,519 | 0,0022 | 0,650 | **10,16** | **5,88** | 0,851 | 0,789 | 0,84 | 0,437 | 0,33 | 0,559 | 0,557 | 0,00865 | 0,614 |
|  | **18 ^b^** | **4,97** | **3,05** | 0,394 | 0,790 | 0,860 | 0,59 | 0,578 | 0,622 | 0,642 | 0,0065 | 0,697 | **6,83** | **3,45** | 1 | 1 | 1 | 1 | 1 | 1 | 1 | 1 | 1 |
|  | **19** | **9** | **6,72** | 0,830 | 0,669 | 0,750 | 0,68 | 0,673 | 0,654 | 0,667 | 0,0011 | 0,644 | **10,47** | **5,22** | 0,763 | 0,643 | 0,79 | 0,567 | 0,43 | 0,601 | 0,461 | 0,00785 | 0,669 |
|  | **20** | **5,64** | **3,98** | 0,903 | 0,702 | 0,790 | 0,673 | 0,572 | 0,681 | 0,667 | 0,0023 | 0,674 | **7,37** | **4,45** | 0,889 | 0,505 | 0,9 | 0,834 | 0,49 | 0,798 | 0,594 | 0,00265 | 0,789 |
| **21-30 days** | **21** | **4,43** | **3,64** | 1,025 | 1 | 0,870 | 0,794 | 0,674 | 0,642 | 0,552 | 0,0034 | 0,674 | **9,24** | **7,45** | 0,923 | 0,430 | 0,88 | 0,234 | 0,51 | 0,771 | 0,67 | 0,0065 | 0,695 |
|  | **22** | **5,55** | **2,42** | 0,859 | 0,986 | 0,792 | 0,811 | 0,651 | 0,619 | 0,431 | 0,0068 | 0,574 | **9** | **6,32** | 0,813 | 0,649 | 0,95 | 0,789 | 0,54 | 0,876 | 0,596 | 0,0056 | 0,684 |
|  | **23** | **5,56** | **1,55** | 0,860 | 0,972 | 0,762 | 0,712 | 0,672 | 0,633 | 0,551 | 0,0023 | 0,728 | **9,43** | **7,9** | 0,842 | 0,977 | 0,87 | 0,89 | 0,59 | 0,756 | 0,618 | 0,0079 | 0,665 |
|  | **24** | **3,44** | **2,76** | 0,829 | 0,827 | 0,870 | 0,789 | 0,452 | 0,701 | 0,441 | 0,0032 | 0,798 | **3,33** | **0,98** | 0,862 | 0,850 | 0,67 | 0,784 | 0,65 | 0,654 | 0,55 | 0,0076 | 0,616 |
|  | **25** | **8,43** | **4,95** | 0,887 | 0,792 | 0,820 | 0,714 | 0,569 | 0,774 | 0,51 | 0,0011 | 0,560 | **11,55** | **7,47** | 0,965 | 0,949 | 0,45 | 0,876 | 0,61 | 0,731 | 0,498 | 0,0043 | 0,641 |
|  | **26** | **9,47** | **5,49** | 0,848 | 0,712 | 0,840 | 0,786 | 0,459 | 0,687 | 0,493 | 0,0056 | 0,791 | **9,7** | **3,35** | 0,915 | 0,813 | 0,86 | 0,98 | 0,64 | 0,762 | 0,573 | 0,0089 | 0,741 |
|  | **27** | **9,71** | **6,62** | 0,840 | 0,777 | 0,762 | 0,763 | 0,568 | 0,653 | 0,594 | 0,0076 | 0,632 | **7,86** | **3,62** | 0,858 | 0,762 | 0,9 | 0,78 | 0,632 | 0,754 | 0,551 | 0,0022 | 0,773 |
|  | **28** | **3,72** | **2,92** | 0,821 | 0,796 | 0,750 | 0,719 | 0,578 | 0,787 | 0,426 | 0,0075 | 0,643 | **9,61** | **5,21** | 0,896 | 0,993 | 0,86 | 0,654 | 0,49 | 0,652 | 0,437 | 0,0068 | 0,715 |
|  | **29** | **2,59** | **1,5** | 0,851 | 0,672 | 0,763 | 0,762 | 0,542 | 0,742 | 0,316 | 0,0012 | 0,704 | **8,22** | **2,56** | 0,893 | 0,643 | 0,85 | 0,621 | 0,59 | 0,741 | 0,512 | 0,0054 | 0,682 |
|  | **30** | **8,63** | **6,64** | 0,593 | 0,609 | 0,712 | 0,678 | 0,543 | 0,713 | 0,507 | 0,0022 | 0,599 | **10,73** | **5,66** | 0,939 | 0,704 | 0,79 | 0,87 | 0,61 | 0,795 | 0,428 | 0,0067 | 0,673 |

^a^ Calibrator strain used for relative quantification of *FUM* genes in 2B710 Hx hybrid;

^b^ Calibrator strain used for relative quantification of *FUM* genes in 2B710 hybrid.
